# Bayesian Spatial Conditional Logistic Regression Modelling of Overweight/Obesity Prevalence and Determinants Among Women in Ghana, Accounting for Contraceptive Use

**DOI:** 10.1155/ghe3/3074676

**Published:** 2026-07-29

**Authors:** Killian Asampana Asosega, Eric N. Aidoo, Atinuke Olusola Adebanji, Ellis Owusu-Dabo

**Affiliations:** ^1^ Department of Statistics and Actuarial Science, Kwame Nkrumah University of Science and Technology, Kumasi, Ghana, knust.edu.gh; ^2^ Department of Mathematics and Statistics, University of Energy and Natural Resources, Sunyani, Ghana, uenr.edu.gh; ^3^ School of Mathematical and Computer Sciences, Heriot-Watt University, Dubai, UAE, hw.ac.uk; ^4^ Department of Statistics, Purdue University, Indianapolis, Indiana, USA, purdue.edu; ^5^ Department of Global and International Health, Kwame Nkrumah University of Science and Technology, Kumasi, Ghana, knust.edu.gh

**Keywords:** Bayesian model, conditional logistic regression, overweight and obesity, spatial model, Sustainable Development Goal 3

## Abstract

**Introduction:**

Overweight/obesity (OW/OB) poses a major public health challenge among reproductive‐aged women across most LMICs, including Ghana. The continuous rise in maternal average BMI coupled with fragile healthcare system makes surveillance and interventions to reverse the trend in Ghana as well as most sub‐Saharan African (SSA) countries, a pertinent issue.

**Objective:**

This study identifies risk factors associated with OW/OB among reproductive‐aged women in Ghana, accounting for spatial autocorrelation and the potential confounding effect of modern contraceptive use (MCU). It also provides updated information on the distribution of OW/OB risk. MCU was used as a stratification variable to control for potential confounding related to contraceptive‐induced metabolic or behavioural influences.

**Methods:**

The recent cross‐sectional Ghana Demographic and Health Survey (2022 GDHS) data, made up of 7054 women aged 15–49 years, was used in this study. The Bayesian spatial conditional logistic regression model was developed to determine significant factors.

**Results:**

About 36.9% of the women were overweight/obese (OW = 22.5% and OB = 14.4%). A significantly high maternal OW/OB rate (> 50) was observed in the Ashanti and Greater Accra regions and the least prevalence (< 20%) in the North‐East region. Regional disparity rates indicate that middle and southern Ghana are associated with higher burden compared to northern Ghana. The Bayesian conditional logistic model with convoluted CAR prior emerged as the best model for studying maternal OW/OB risk while accounting for MCU with the minimum WAIC and DIC values. Significantly higher risk of OW/OB burden is linked to high education (primary [aOR = 1.87, 95% CI:2.27–6.50]; secondary [aOR = 2.02, 95% CI: 2.42–7.60]; and higher [aOR = 2.05, 95% CI: 1.59–2.64]), increase in age (20–29 [aOR = 3.34, 95% CI: 2.67–4.17]; 30–39 [aOR = 6.07, 95% CI: 4.66–7.89]; and 40–49 [aOR = 7.22, 95% CI: 5.40–9.65]), being married (aOR = 1.81, 95% CI: 1.49–2.19), urban residency (aOR = 1.41, 95% CI: 1.23–1.61) and the number of children (aOR = 1.06, 95% CI: 1.03–1.11).

**Conclusion:**

The spatial heterogeneity in OW/OB risk provided evidence for regional targeted preventive strategies, particularly in the Ashanti and Ahafo regions, which are significant hotspots for high OW/OB risk, to help reduce the public health burden of maternal OW/OB in Ghana. This could contribute to ensuring the quality health of women (SDG 3).

## 1. Introduction

Overweight and obesity (OW/OB) prevalence has witnessed significant increase in recent decades, especially in low‐ and middle‐income countries (LMICs) [1–3]. The continuous rise in OW/OB prevalence among reproductive‐aged women poses significant public health concerns [4–6]. OW/OB is common risk condition for chronic and noncommunicable diseases (NCDs) [7, 8]. NCDs associated with OW/OB account for at least five million deaths worldwide, with more than 75% of these deaths occurring in LMICs [9]. Reproductive‐aged women are seriously affected by the consequences of OW/OB particularly during pregnancy and childbirth. OW/OB is linked to adverse health outcomes in mothers and their foetuses [10–13].

OW/OB was previously considered public health problems primarily affecting high‐income countries (HICs). However, recent trends indicate that they are now prevalent in most developing countries particularly in sub‐Saharan Africa (SSA) [14, 15]. The prevalence varies significantly across SSA, with South Africa, Mauritania and Eswatini reporting the highest rates [1]. In addition, the prevalence of OW/OB has been found to vary across different geographical areas and population groups in many countries including Ghana [16–18] and Nigeria [19]. Urban areas are consistently associated with higher prevalence of OW/OB compared to rural regions [2, 3]. A higher risk of OW/OB has also been observed among women who are highly educated, from wealthier households, older in age and multiparous [19–21].

While several factors have been identified as influencing the risk of OW/OB, an individual’s contraceptive use status may act as a confounder in the relationship between these factors (e.g., sociodemographic status) and OW/OB status [16, 17]. For instance, while some literature has established a relationship between women’s socioeconomic status and their contraceptive use [18, 22], others have found a positive relationship between contraceptive use and OW/OB status [23]. Modern contraceptive use (MCU) among reproductive‐aged women is primarily intended to prevent unintended pregnancies, promote birth spacing, reduce unsafe abortions, mitigate maternal morbidity and mortality, and decrease adverse pregnancy outcomes [24–26]. In pursuit of these goals, governments and development partners have invested resources to promote the uptake of modern contraception, aligning with efforts to achieve Sustainable Development Goals (SDGs) 3 and 5 [27–29]. Despite its numerous benefits for individuals, households and broader society, MCU has been associated with an increased risk of OW/OB among reproductive‐aged women, particularly with prolonged use of certain modern contraception methods [23, 30, 31].

Although evidence on the association between MCU and OW/OB risk is mixed and may vary depending on the type of contraceptive used, it is therefore imperative to account for this relationship when identifying OW/OB risk factors among reproductive‐aged women. In this study, the confounding effect of contraceptive use on OW/OB analysis was accounted for using a novel Bayesian approximate spatial conditional logistic regression (CLR) model. The conventional CLR is a statistical technique used to analyse matched or stratified data with binary outcomes [32, 33]. The CLR model has received numerous applications across diverse disciplines [34–38]. Despite the extensive literature on the use of CLR modelling in matched case–control studies, there is limited literature on studies that account for spatial components into CLR models to explain spatial variations in the outcome variable. Thus, investigating factors influencing OW/OB while accounting for spatial autocorrelation and the confounding effect of contraceptive use among reproductive‐aged women will not only inform and update stakeholders on the current OW/OB situation among reproductive‐aged women but also help identify areas with high risk of OW/OB and provide insights to enhance targeted public health interventions. These may include efficient allocation of resource allocation, increased surveillance and regional tailored public health education to curtail the public health burden associated with high OW/OB in Ghana.

## 2. Materials and Methods

### 2.1. Data and Sampling

The cross‐sectional nationally representative data from the recent 2022 Ghana Demographic and Health Survey (2022 GDHS) were used for this study. The 2022 GDHS as part of the seventh series of the DHS Program provides extensive secondary data collected using probability sampling techniques and internationally accepted standard protocols [39, 40]. A two‐stage sampling procedure was implemented in the nationwide survey. This involved the initial selection of 618 enumeration areas, followed by the random selection of households and reproductive‐aged women (15–49 years) eligible for interview across the 16 regions of Ghana. An average of 30 households were randomly selected from each enumeration area which constituted a total of 18, 450 households for the 2022 survey. In all, a total of 15,014 reproductive‐aged women were randomly obtained from the sampled households. For further details on the design of GDHS, refer to https://www.dhsprogram.com/Methodology/Survey-Methodology.cfm.

### 2.2. Study Sample and Variables

From the initial 15,014 reproductive‐aged women interviewed from the 18,450 household randomly selected for the 2022 GDHS, those without height/weight measurements (7,397), pregnant at the time (559) and flagged cases (4) were excluded from the analysis. The final study sample of 7054 reproductive‐aged women with complete information was used in the statistical analysis.

In this study, the outcome variable of interest was the OW/OB status, which was determined using the body mass index (BMI) of reproductive‐aged women. The BMI classification for each of the final 7054 women is based on their anthropometric measurements (weight and height). Reproductive‐aged women with BMI ≥25 *k* *g* *m* ^−2^ were considered OW/OB coded ‘1’, while those with BMI <25 *k* *g* *m* ^−2^ were considered non‐OW/OB and coded ‘0’ in accordance with the WHO classification [41]. The possible covariates considered in this study are maternal age (< 20, 20–29, 30–39 or 40–49), marital status (single, cohabiting, married and others [separated/divorced/widowed]), educational attainment (no formal education, primary, secondary or higher education), type of place of residence (rural and urban), household wealth category (poor, middle or rich), gender of household head (male or female), household head age and the region of residence. The MCU variable was classified as either ‘use’ or ‘non‐use’ among individual women.

### 2.3. Conventional CLR Model

In this study, a CLR model was used to examine the relationship between OW/OB (the outcome) and the various sociodemographic factors while controlling the confounding effect of MCU. The CLR model is commonly used to assess the association between sociodemographic factors and outcome in a stratified or matched data setting [38, 42]. In this study, MCU was included in the model as a stratification variable to control its confounding effect on the relationship between OW/OB and the sociodemographic factors without requiring individual matching. That is, individuals were grouped based on their contraceptive use status to allow the model to focus on within‐group comparison.

Suppose *n* individuals are divided into *J* strata (in this case, two groups: contraceptive users and nonusers), where each stratum consists of both OW/OB (*y*_*i* *j*_ = 1) and non‐OW/OB (*y*_*i* *j*_ = 0). Also, let *j* ∈ {1,  . . . ,  *J*} index the strata and *i* ∈ {1,  . . . ,  *n*} index the individuals, the CLR model for an individual *i* in stratum *j* who is OW/OB is defined as follows [32]:

(1) logPyij=1xij,β=μj+xijTβ,

where *μ*_*j*_ represents fixed intercept defined for specific stratum, *P*(*y*_*i* *j*_|*x*_*i* *j*_) represents the probability that an individual *i* in stratum *j* is OW/OB (*y*_*i* *j*_) conditioned on a vector of covariates *x*_*i* *j*_ for individual *i* in stratum *j*, while *β* represents a vector of regression coefficients associated with the covariates. For each stratum *j*, we want to condition on the stratum and estimate the parameters *β* based on the relative odds of being a case versus a control within each stratum. The conditional likelihood function for the stratified data is constructed by considering the likelihood for all strata. For each stratum *j*, the conditional likelihood function of the outcomes is given by:

(2) Ljβ=exp  xijTβ∑k=1njexp  xjkTβ,

where *k* ∈ {1,  . . . , *n*_*j*_ } runs over all individuals in stratum *j*. The stratum‐specific intercept term cancelled out in the construction of the conditional likelihood function [32, 43]. The conditional likelihood function for the entire data set is the product of the likelihood function across all strata:

(3) Lβ=∏j=1Jexp  xijTβ∑k=1njexp  xjkTβ.

In the CLR model, the likelihood is conditioned on the stratified variable (i.e. contraceptive usage in this case), such that the model only focuses on the within‐stratum group variation, rather than between different stratum groups. To further account for the spatial effects in OW/OB risk, the CLR model is extended to capture the spatial effects in addition to the stratification variable (modern contraceptive) through the spatial conditional logistic regression (SCLR) model under the Bayesian framework.

### 2.4. Bayesian Approximate SCLR Model

The conventional CLR model was extended to account for spatial hierarchical structure in the data. The SCLR is utilised and preferred since traditional models usually require independent observations, which is insufficient for the DHS data for this study. The proposed SCLR model accounts for spatial dependency—the phenomenon where women in proximity are likely to share similar infrastructure, food environments and regional cultural norms. Let *y*_*s* *i* *j*_ represents the binary outcome for individual *i* in stratum *j* at location *s* ∈ {1,  . . . , *S* }, *x*_*i* *j*_ represents the corresponding covariates for the individual, then the SCLR model is defined as follows:

(4) logPysij=1xsij,β,μj,θs=μj+xsijTβ+θs,

where *μ*_*j*_ is the random intercept for stratum *j* and accounts for unobserved heterogeneity that vary across different stratum, while *θ*_*s*_ represents the spatial random effect for location *s* shared by all individuals in that location. The conditional likelihood function for the SCLR model is obtained by summing all observations within the stratum *j* defined as follows:

(5) Ljβ,μ,θ=exp  μj+xsijTβ+θs∑k=1njexp  μj+xsjkTβ+θs.

The full likelihood function which combines the likelihood functions for the entire dataset at all locations is given by

(6) Lβ,μ,θ=∏j=1Jexp  μj+xsijTβ+θs∑k=1njexp  μj+xsjkTβ+θs.

Given the full likelihood function for the data, the parameters for the model were estimated using the integrated nested Laplace approximation (INLA) in the Bayesian framework [44, 45]. The Bayesian INLA approach is considered to be much faster than the conventional Markov chain Monte Carlo (MCMC) methods when dealing with data with hierarchical structure such as the data used in this study. The implementation of the Bayesian INLA approach requires prior distribution for all the parameters in order to approximate their posterior distributions.

The random intercept *μ*_*j*_ was assumed to follow a normal distribution with mean 0 and variance $\sigma_{\mu }^{2}\left(P\left(\left.\mu \right|\sigma_{\mu }^{2}\right)∼\;N\left(0,\;\sigma_{\mu }^{2}\right)\right)$. The prior distribution for the fixed‐effect coefficient *β* was assumed to follow a normal distribution with mean 0 and variance $\sigma_{\beta }^{2}$ $\left(P\left(\left.\beta \right|\sigma_{\beta }^{2}\right)∼\;N\left(0,\;\sigma_{\beta }^{2}\right)\right)$. The spatial random effect component *θ*_*s*_ was modelled using conditional autoregressive (CAR) priors which satisfies the conditions of Markov random fields [46]. The CAR prior distributions considered in this study include the intrinsic CAR (ICAR), convoluted CAR and Leroux conditional autoregressive (LCAR) models [46, 47]. The ICAR model assumes that the spatial random effects at each location are conditionally dependent on those of its neighbouring locations. The ICAR model can be written as follows:

(7) θsθ−s ∼ N∑m=1Swsmθm∑m=1Swsm,σ2∑m=1Swsm,

where *θ*_−*s*_ = {*θ*_1_, …, *θ*_*S*−1_, *θ*_*s*+1_, …, *θ*_*S*_} denotes a vector of locations that is spatially adjacent (or close) to location *s*, *w*_*s* *m*_ are the weights for the spatial connections between location *s* and *m* such that *w*_*s* *m*_ = 1 if *s* and *m* are adjacent area neighbours and 0 otherwise, while $\sigma_{\theta }^{2}$ is the variance of the spatial random effects. The conditional expectation of *θ*_*s*_ is equal to the average of the random effects in neighbouring areas, while the conditional variance is inversely proportional to the number of neighbours.

The convoluted conditional autoregressive (CCAR) model proposed by Besag, York and Molli´e (BYM) extends the conventional ICAR model to incorporate independent random component (unstructured spatial component) into the ICAR framework [48]. This model is particularly useful when the spatial dependency structure is more complex or when the data exhibit heterogeneity across regions that are not easily captured by simple autoregressive processes [49]. In the CCAR model, the spatial random effect component *θ*_*s*_ is partition into two components: *θ*_*s*_ = ∅_*s*_ + *φ*_*s*_, where ∅_*s*_ and *φ*_*s*_ represent the spatially structured and unstructured random effect, respectively. The component ∅_*s*_ is equal to *θ*_*s*_ in the ICAR specification, while the spatially unstructured random effect *φ*_*s*_ was modelled independently for each spatial location using a normal prior with mean 0 and variance $\sigma_{\varphi }^{2}$ $\left[\varphi_{s}∼\;N\left(0,\;\sigma_{\varphi }^{2}\right)\right.$]. The spatially unstructured random effect *φ*_*s*_ accounts for area‐specific random noise.

The LCAR model, however, extends the CAR model to account for both local dependencies and a global spatial correlation effect [50]. It uses a partial sill parameter to model varying degrees of spatial correlation across the spatial locations to capture spatial heterogeneity. However, it is computationally more demanding and may require careful interpretation due to its hierarchical structure and additional parameters. The LCAR model for the spatial random effects can be written as follows:

(8) θsθ−s ∼ Nρ∑m=1Swsmθmρ∑m=1Swsm+1−ρ,σ2ρ∑m=1Swsm+1−ρ,

where 0 ≤ *ρ*  <  1 represents the spatial autocorrelation parameter whose prior is assumed to be uniformly distributed (*ρ* ~  *U*(0,  *M*_*ρ*_). The parameter *ρ* controls the degree of spatial autocorrelation such that *ρ* = 0 indicates independence and a value increasing towards 1 indicates stronger autocorrelation. For *ρ* = 1, the LCAR model converges to the ICAR model.

The models capture both the uncorrelated and correlated spatial dependencies in the data, while adjusting for covariates and random intercepts within the stratum. The posterior distribution of the model parameters is proportional to the product of the full likelihood function of the entire dataset and the prior distributions for the random effect given by

(9) πβ,μ,θ,σμ2,σβ2,σθ2y,x∝Lβ,μ,θY,X×Pμσμ2×Pβσβ2×Pθσθ2,

where $\sigma_{\mu }^{2},\sigma_{\beta }^{2}$ and $\sigma_{\theta }^{2}$ represent hyperparameters and are estimated in addition to the model parameters. In the case of CCAR and LCAR model, two additional hyperparameters ($\sigma_{\varphi }^{2}\;andM_{\rho }$) were estimated. To avoid shrinkage effect on the random intercept term, the variance $\sigma_{\mu }^{2}$ was fixed at large value, 10^6^ [51]. For efficient computation, a precision parameter *τ* = *σ* ^−2^ is used instead of the variance parameters. The precision parameters *τ*_*β*_ and *τ*_*θ*_ were assumed minimal noninformation prior [logτ ~ logGamma(1,  0.0005)]. The Bayesian framework through INLA allows us to estimate the model parameters efficiently, and the variants of the SCLR models were compared by means of the deviance information criterion (DIC) and the widely applicable information criterion (WAIC). The model with the smallest DIC and WAIC is considered the most suitable model for the data. Although both criteria aim to balance between model fit and complexity, the WAIC provides more robust and accurate estimation of predictive performance particularly for complex and hierarchical models.

## 3. Results

The prevalence rate of OW/OB among the reproductive‐aged women in Ghana was about 37% (Table 1). The OW/OB prevalence among reproductive‐aged women increased with advancing in age, with rates of 9.8%, 32%, 50.1% and 53% among women aged less < 20, 20–29, 30–39 and 40–49 years, respectively. More than half (54.9%) of reproductive‐aged women with high educational attainment were OW/OB, compared to 38.4%, 36.3% and 26.5% of those with secondary, primary and no formal education being OW/OB, respectively. Women from the rich and middle wealth bracket households were associated with high OW/OB rates of 55.3% and 40.0%, respectively, compared to their counterparts from poor (21.8%) households. Marital status was another factor for OW/OB among reproductive‐aged women. Most (55%) of women who were divorced, widowed or separated were OW/OB. Similarly, 42.9% and 44.7% of women who were married and cohabiting, respectively, were OW/OB, compared to 21.7% among single women. Furthermore, a higher proportion (46.7%) of urban‐resident women was classified as OW/OB relative to 27.2% of rural‐resident women. Among women with 4–6 children, 46.5% were OW/OB, followed by 43.8% of those with 1–3 children and 41.3% of women with seven or more children. Additionally, 44.3% of women who use modern contraceptives were OW/OB, compared to 34.4% among nonusers. The *p* value of the chi‐square test suggests a significant relationship between OW/OB status and the other variables at 5% alpha level, except the age of household head.

**TABLE 1:** Percentage (%) distribution of OW/OB among reproductive‐aged women in Ghana, classified by the other variables considered in the study, along with chi‐square test *p* value.

Variable	Total	OW/OB (%)	Non‐OW/OB (%)	*p*‐value
Total	7054	36.900	63.100	
Maternal age				< 0.001
< 20	1397	9.800	90.200	
20–29	2259	32.000	68.000	
30–39	1955	50.100	49.900	
40–49	1443	53.000	47.000	
Education attainment				< 0.001
No education	1550	26.500	73.500	
Primary	1051	36.300	63.700	
Secondary	3843	38.400	61.600	
Higher	610	54.900	45.100	
Wealth status				< 0.001
Poor	3226	21.800	78.20	
Middle	1426	40.000	60.00	
Rich	2402	55.300	44.70	
Marital status				< 0.001
Single	2417	21.700	78.300	
Married	3142	42.900	57.100	
Cohabiting	866	44.700	55.300	
Divorced/widowed/separated	629	55.000	45.000	
Place of residence				< 0.001
Rural	3543	27.200	72.800	
Urban	3511	46.700	53.300	
Number of children				< 0.001
None	2254	20.700	79.300	
1–3	2916	43.800	56.200	
4–6	1601	46.500	53.500	
7+	283	41.300	58.700	
Modern contraceptive use				< 0.001
No	5284	34.400	65.600	
Yes	1770	44.300	55.700	
Gender of household head				< 0.001
Female	2797	42.400	57.600	
Male	4257	33.310	66.690	
Age of household head				0.457
< 20	34	38.240	61.760	
20–29	884	38.460	61.540	
30–39	1799	37.470	62.530	
40–49	2052	36.350	63.650	
50–59	1038	38.730	61.270	
60–69	854	35.130	64.870	
70–79	266	33.830	66.170	
80–89	99	32.320	67.680	
90–99	28	25.000	75.000	

**FIGURE 1:**
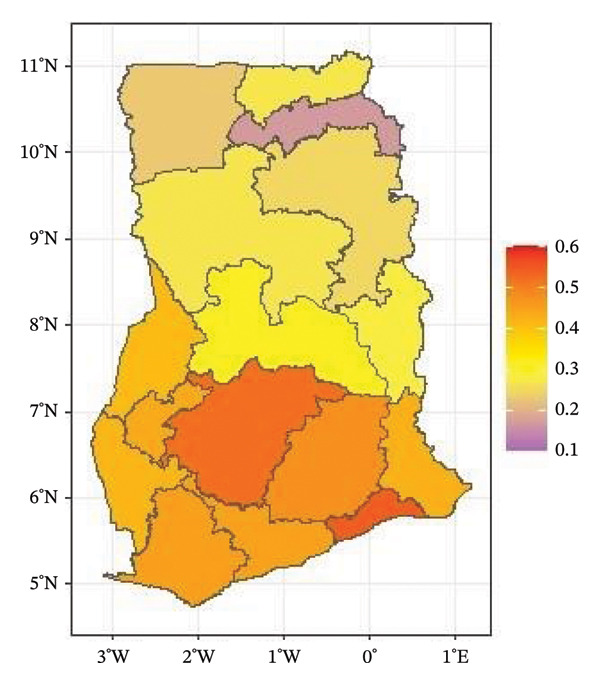
Spatial distribution of overweight/obesity proportion across Ghana (raw prevalence).

The spatial distribution of the OW/OB prevalence rate showed that OW/OB was not uniformly spread across the 16 regions of Ghana (Figure 1). Higher proportions of OW/OB were clustered in the southern parts of Ghana, while relatively lower clustering was observed in the northern parts (Figure 1). The spatial clustering highlights the need to incorporate the geographical dimension into OW/OB risk modelling, in order to provide more insights into its prevalence and underlying dynamics.

To account for sociodemographic and spatial random effects in assessing the risk of OW/OB, four different Bayesian approximate SCLR models were specified. These comprised a non‐SCLR model, along with three models that incorporated different variants of spatial random effect components. During model specification, MCU was incorporated as a stratification variable to control for its potential confounding effect on the relationship between OW/OB status and the sociodemographic factors. The performance of the four models described by the WAIC and DIC is summarised in Table 2. Based on the comparison metrics, the spatial model incorporating a convoluted CAR prior outperforms the other three models. Although the differences in the WAIC and DIC among the spatial models are marginal, our choice of the convoluted CAR model over the other was also guided by both theoretical and practical consideration in addition to the model fit criteria. For instance, the convoluted CAR model decomposes the spatial random effect into both structured (spatial autocorrelation) and unstructured (spatial random noise) components [48]. This feature provides interpretability in understanding regional variations which was important in the OW/OB analysis. In contrast, the ICAR model assumes that the spatial random effect is spatially structured, which may be too restrictive in our context where some variations may be due to unmeasured effect in the regions including population densities, or administrative practices. The Leroux, however, uses a single parameter to control the structured and unstructured random effect [50], but the parameter has no direct interpretation. Thus, the choice of the convoluted CAR model was sufficiently justified.

**TABLE 2:** Model comparison metrics.

Spatial model type	WAIC	DIC
IID (no spatial model)	7574.115	7573.789
Intrinsic CAR model	7522.538	7522.075
Convoluted CAR model	7522.511	7522.016
Leroux CAR model	7522.875	7522.365

The posterior means and other parameter characteristics from the selected Bayesian approximate SCLR model with the convoluted CAR prior are presented in Table 3. The result of the model highlights the significant contribution of the spatial components to the risk of OW/OB among reproductive‐aged women in Ghana. In addition, the influence of the individual and household characteristics of reproductive‐aged women on OW/OB risk was assessed and discussed. The 95% Bayesian credible interval suggests that all the sociodemographic variables were statistically significant except the household head age.

**TABLE 3:** Posterior mean, standard deviation (SD), 95% Bayesian credible interval (BCrI) and the adjusted odds ratios (aOR) of the parameter of the Bayesian approximate spatial conditional logistic regression model with convoluted CAR prior.

Variable	Mean	SD	95% BCrI		aOR
			2.5%	97.5%	
Fixed effects					
Intercept	−3.78	707.1	−1390.336	13.828	
Maternal age (Ref: < 20)					
20–29	1.205	0.114	0.982	1.428	3.337
30–39	1.803	0.134	1.540	2.065	6.068
40–49	1.977	0.148	1.687	2.267	7.221
Marital status (Ref: Single)					
Married	0.591	0.098	0.399	0.782	1.806
Cohabiting	0.544	0.107	0.334	0.753	1.723
Others (separated/divorced/widowed)	0.581	0.120	0.345	0.817	1.788
Educational attainment (Ref: No formal education)					
Primary	0.627	0.102	0.427	0.827	1.872
Secondary	0.707	0.090	0.531	0.883	2.028
Higher	0.718	0.129	0.466	0.971	2.050
Number of children	0.066	0.020	0.028	0.104	1.068
Wealth status (Ref: Poor)					
Middle	0.621	0.085	0.454	0.788	1.861
Rich	1.167	0.089	0.993	1.342	3.212
Residence (Ref: Rural)					
Urban	0.344	0.068	0.210	0.477	1.411
Gender of household head (*Ref*: Female)					
Male	−0.245	0.065	−0.373	−0.116	0.783
Age of household head (Ref: < 20)					
20–29	0.155	0.405	−0.639	0.950	1.168
30–39	0.143	0.401	−0.644	0.929	1.154
40–49	0.086	0.400	−0.699	0.871	1.090
50–59	0.175	0.404	−0.617	0.966	1.191
60–69	0.012	0.405	−0.783	0.807	1.012
70–79	0.026	0.423	−0.804	0.857	1.026
80–89	−0.006	0.467	−0.922	0.910	0.994
90–99	−0.099	0.652	−1.378	1.180	0.906
Random effects					
Spatially unstructured effect	2279.55	2452.93	165.73	8793.51	
Spatially structured effect	15.45	8.26	5.05	13.59	

The results from the model suggest that the likelihood of OW/OB was positively related to the age of the women. That is, women aged 20–29, 30–39 and 40–49 years had approximately 3, 6 and 7 times higher odds, respectively, of being OW/OB compared to those under 20 years. Similarly, the odds of being OW/OB for married, cohabiting and other (divorced/widowed/separated) women were 1.8, 1.7 and 1.8 times higher, respectively, compared to single women. With respect to educational attainment, it was found that women with higher education had higher odds of being OW/OB. Specifically, women with primary, secondary and higher education had 1.8, 2.0 and 2.1 times higher odds, respectively, of being OW/OB compared to those with no formal education. An increase in the number of children ever born to a woman was associated with 1.1 times higher odds of being OW/OB. The likelihood of OW/OB was positively associated with increasing household wealth status. Specifically, women from household with middle and rich wealth status had 1.8 and 3.2 times higher odds, respectively, of being OW/OB compared to those from poor household. In the case of urbanisation, the study results show that the odds of being OW/OB were 1.4 times higher for women in the urban areas compared to those in the rural areas.

The gender of household heads was significantly associated with OW/OB risk among reproductive‐aged women. The results show that women from male‐headed households had 0.78 times lower odds of being OW/OB compared to those from female‐headed households. The age of household head was not significantly associated with OW/OB in Ghana. However, an increase in the age of the household head was associated with higher odds of OW/OB among reproductive‐aged women.

**FIGURE 2:**
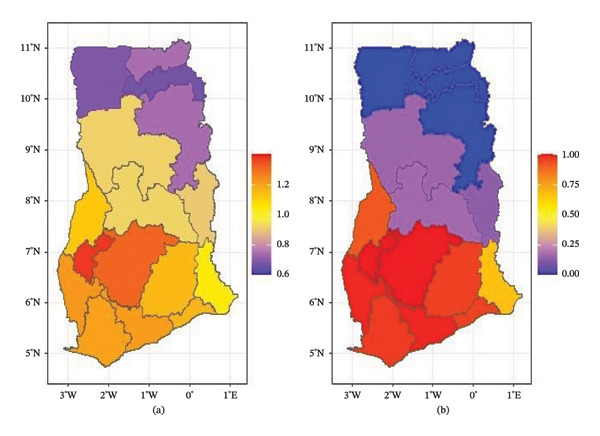
Spatial distribution of the posterior mean of regional‐specific odds ratios (posterior odds ratios) (a) and the associated posterior probability of exceeding one (b) based on the selected spatial model.

The posterior means of the regional‐specific odds ratios indicate notable regional disparities in OW/OB risk for reproductive‐aged women in Ghana (Figure 2a). This spatial clustering underscores the potential influence of neighbourhood effects on OW/OB risk, consistent with the first law of geography which says that nearby observations are more similar than distant observations. The likelihood of OW/OB among reproductive‐aged women in Ghana was significantly higher in the Ahafo region, followed by the Ashanti, Central, Western North and Western regions. In contrast, the Upper West, Upper East, North‐East and Northern regions showed lower likelihood of OW/OB risk. The exceedance probability analysis further confirms a high likelihood of OW/OB risk in the southern part of Ghana with the highest risk than in the northern part (Figure 2b). The variance inflation factors for all independent variables considered for modelling were less than 3, suggesting no evidence of multicollinearity (Table 4).

**TABLE 4:** Variance inflation factor (VIF) for model independent variables.

Variable	VIF
Maternal age	2.580
Marital status	2.447
Education attainment	1.791
Number of children	2.638
Wealth status	1.790
Place of residence	1.407
Gender of household head	1.272
Age of household head	1.017

## 4. Discussion

The prevalence of OW/OB among reproductive‐aged women has increased significantly globally over the past decade, with particularly alarming rates observed in LMICs compared to HICs [52, 53]. In the present study, the OW/OB prevalence of 36.9% among reproductive‐aged women in Ghana highlights the significant health consequences for both women and their offsprings [10, 54]. This figure is 1.4% higher than the 35.5% prevalence reported among reproductive‐aged women in Ghana based on the 2014 GDHS data [40]. The OW/OB prevalence observed in this current study is higher than the pooled prevalence of 27% reported for 23 countries in SSA excluding Ghana, which ranged from 7.4% in Ethiopia to 49.7% in Mauritania [52]. Similarly, Tamir et al. [55] also observed a pooled OW/OB prevalence of 34.8% across 9 SSA countries including Ghana. In the same study by Tamir and colleagues [55], OW/OB was more prevalent among reproductive‐aged women in Gabon (48.3%) and less prevalent in Mozambique (23.4%). Furthermore, the OW/OB prevalence in this study exceeded the 27.4% reported among reproductive‐aged women in Nigeria [56]. However, it remains lower than the 61.6% reported among South African women [4] and the 49.5% prevalence of OB alone among Egyptian women [57]. Although OW and OB rates have risen alarmingly across the African content, the Northern and Southern regions are the most affected [2, 52, 58]. The potential risk factors for OW/OB were identified using four different Bayesian approximate SCLR models. These comprised a non‐SCLR model, along with three models that incorporated different variants of spatial random effect components. During model specification, MCU was included as a stratification variable to control for its potential confounding effect on the relationship between OW/OB and possible sociodemographic status variables. This decision was informed by emerging evidence suggesting that MCU may be associated with both OW/OB and the sociodemographic factors [16, 23]. Biologically, some hormonal contraceptives have been linked to changes in metabolism, appetite regulation, fluid retention and fat distribution, which may contribute to weight gain in some women [59]. From behavioural perspectives, MCU may correlate with higher educational attainment, access to healthcare services and greater health awareness. These factors are also associated with sociodemographic status and health‐related behaviours including dietary patterns and physical activities [18, 22]. By stratifying on MCU status, the proposed model accounts for the possibility that the relationship between sociodemographic status and the risk of OW/OB may differ based on contraceptive use. This approach helps to capture potential heterogeneity across subgroups, thereby enhancing the internal validity of the findings. The performance of the models based on the WAIC and DIC suggests that the spatial model with convoluted CAR prior better suits the data compared to the other three models considered.

The results from the selected model indicate spatial disparity in the risk of OW/OB. These disparities in OW/OB prevalence among reproductive‐aged women in Ghana reflect the regional inequalities in OW/OB risk. The significant clustering of OW/OB risks highlights the impact of neighbourhood effects on OW/OB risk. These findings align with previous studies [60]. For instance, Yussif et al. [60] reported a significantly high prevalence of OW/OB in southern and middle parts of Ghana compared to the northern parts of Ghana. The continued rise in the prevalence of OW/OB rates among reproductive‐aged women coupled with the spatial clustering across all regions in Ghana further suggests growing burden of adverse health outcomes especially cardiovascular diseases and a decline in overall quality of life [60]. In addition, the disproportionately higher burden of OW/OB among women in urban areas underscores the importance of identifying regional hotspot to support the implementation of targeted public health intervention strategies in Ghana [52].

The high prevalence of OW/OB in Ghana as well as other LMICs mostly in SSA is driven by several factors. However, sedentary lifestyle and nutritional transition are among the primary contributors [61–63]. These factors are exacerbated by increasing urbanisation and modernisation which promotes easy access to fast food and modern food retail sectors coupled with less physical activity [64, 65]. The regional disparities in the OW/OB risk further suggest that the high risks in the Ahafo and Ashanti regions persisting after adjusting for demographic characteristics (wealth and education) could be linked to some unmeasured regional factors, such as cultural perceptions of body size or culinary traditions that contribute to these hotspots. Again, the regional disparity in OW/OB risk among women in this current study could also be attributed to the inequality in level of urbanisation across the 16 regions in Ghana. For instance, the Southern regions particularly Ahafo, Ashanti and Greater Accra regions exhibit higher OW/OB rates than regions in the North, such as the Upper East, Upper West and North‐East regions. These findings are consistent with a systematic review that reported elevated OW/OB rates in southern Ghana [66, 67]. Additionally, many SSA countries have documented a link between higher OW/OB risk and urbanisation [21, 58, 68, 69].

In addition, a significant difference in OW/OB risk was observed between rural and urban dwellers. These findings support previous studies, which consistently report a higher OW/OB burden among urban‐dwelling women compared to their rural counterparts [2, 68]. The urban–rural disparity in OW/OB risk may be attributed to the fact that urban women are more likely to engage in reduced physical activities, access fast foods and consume sugar‐sweetened beverages [2, 65, 70]. This behaviour can lead to an energy imbalance, where the body uses less energy than it consumes, thereby elevating the risk of OW/OB [71, 72].

Beyond the geographical disparities in OW/OB rates among Ghanaian reproductive‐aged women, the results further revealed higher risk of OW/OB among educated women compared to those with no formal education. The increase in the likelihood of OW/OB burden among women with some level of education confirms the findings reported in previous studies across most LMICs [1, 5, 31]. The study also found a higher risk of OW/OB among women from middle‐ and high‐income households compared to those from poorer households. This association between household wealth status and elevated OW/OB risk has been reported in studies from various developing countries [1, 70, 72]. The established positive association between OW/OB burden and higher education level of education and household wealth provides sufficient evidence that women with high socioeconomic status in LMICs, such as Ghana, are more likely to engage in fewer physical activities (sedentary behaviour), coupled with the consumption of energy‐dense and highly processed foods, which increases the risk of OW/OB [73, 74]. This trend is not unique to Ghanaian women but is commonly observed in many LMICs as compared to HICs [75]. Moreover, Ghana as a LMIC is experiencing a nutrition transition, a shift from traditional diets and lifestyles towards more westernised patterns, such as increased consumption of processed foods, sugar‐sweetened beverages and more sedentary lifestyles due to rapid urbanisation and changes in work and transportation. Again, wealthier women can afford energy‐dense, processed food, which are more prestigious and convenient but are high in sugar, calories and fat. Although higher education generally promotes health awareness, in rapidly urbanising settings, it may correlate with less physically active jobs and higher disposal income for unhealthy foods contributing to the obesogenic environment in most urban areas in Ghana [63, 76].

Furthermore, marital status and age were found to significantly correlate with higher odds of OW/OB risk among reproductive‐aged women. Women aged 40–49 years had over seven times the odds of being OW/OB compared to those under 20 years, indicating a strong positive association between an increase in age and OW/OB risk. This positive association between age and high risk of OW/OB has been observed in the literature [1, 58, 61]. For instance, women aged 35–49 years were at least three times more likely to be OW/OB compared to those aged 15–24 years in Ethiopia [77]. Similarly, Mosha et al. [78] reported that women aged 35–49 years in Tanzania were 60% more likely to be OW/OB than those aged 15–24 years. This positive association between age and increased burden of OW and OB may be attributed to reduced physical activeness characterised by old age and the consumption of energy‐dense foods, which may contribute to weight gain and subsequently OW/OB [78–80]. These significantly high odds of OW/OB in women aged 40–49 years compared to young women could be attributed to the potential metabolic effect of parity interacting with the biological ageing of women through physiological stress, persistent weight retention and metabolic shifts [81]. Additionally, the study found a significant association between marital status and OW/OB risk. That is, women who were cohabiting, married and separated/divorced/widowed had higher odds of OW/OB risk compared to single women. This supports the concept of marital status and the attractiveness model of body image, where single women are relatively more concerned about maintaining a desirable body shape to attract potential partners for marriage [82, 83]. Furthermore, women with at least one child were found to have a significantly higher risk of OW/OB than women without children [84]. Despite the relevance of these findings, there is potential collinearity between parity and increase in maternal age masking the risk of OW/OB among women. Previous studies have shown that older women are more likely to have higher parity, and higher parity is significantly linked to high risk of OW/OB [85]. However, earlier findings have also established that higher parity and advancing in age independently increase the risk of OW/OB among reproductive‐aged women [40, 86].

The findings of the study are not without limitations due to the exclusion large number of records for women with missing anthropometric data (height and/or weight measurements), which constitute nearly half the total sample. Since the missing data were not completely at random, the final sample analysed may differ systematically from the full sample, potentially introducing selection bias. Although the Bayesian approach used provides a robust inference for the analysed sample and accounts for potential uncertainty in the model parameters, it does not fully address the issue of selection bias. Thus, the generalisability of the findings of the study may be affected. Future studies should explore whether the excluded responses significantly differ in a way that could bias the observed relationship between OW/OB risk and the sociodemographic factors. Again, this present study did not distinguish between hormonal and nonhormonal contraceptive use among reproductive‐aged women. Given that weight gain is biologically linked primarily to hormonal methods, this broad grouping may mask more significant effects.

## 5. Conclusion and Policy Implications

OW/OB rates among reproductive‐aged women in Ghana, as well as in most LMICs, remain high and on the rise with significant adverse health outcomes. The burden of OW/OB among reproductive‐aged women is comparatively higher in the southern and middle parts of Ghana than in the northern parts. In this study, a Bayesian approximate spatial conditional logistic model was proposed and applied to identify risk factors for OW/OB among reproductive‐aged women in Ghana. The study contributes to the literature by using a model which does not only account for the spatial random effect but also account for the confounding effect of MCU on the relationship between OW/OB and sociodemographic factors. In addition, the use of the Bayesian approximate inference provides full posterior distribution of model parameters allowing for uncertainty quantification which are very useful for survey data.

The Bayesian spatial model with convoluted CAR prior was found to be suitable for the data. These findings further suggest that the Bayesian variant of the conditional logistic model employed in this study is a promising tool in providing insightful information on OW/OB risk among reproductive‐aged women. The results revealed that marital status, maternal age, higher educational attainment, high household wealth and urban residency were positively associated with increased risk of OW/OB. Additionally, there was significant spatial disparity in the risk of OW/OB across regions in Ghana. The future outlook of the prevalence of OW/OB further suggests a looming public health burden of OW/OB among reproductive‐aged women. The spatial/regional disparity in the risk of OW/OB, coupled with the significant demographic factors among reproductive‐aged women, should guide the implementation of urgent and targeted preventive strategies to reduce the burden of OW/OB among reproductive‐aged women within the study setting and other developing countries, particularly within SSA.

The spatial heterogeneity in OW/OB risk suggests the need for regional‐specific prevention strategies, such as urban food environment regulation to control the marketing of ultraprocessed foods and mass media campaigns in local languages on the importance of being physically active and weight management, particularly urban areas in the Ashanti and Ahafo regions. Since maternal age and marital status were identified as high‐risk factors, policies should integrate routine screening and nutritional counselling into healthcare services, with specific attention given to older and married women who may be at high‐risk. Additionally, educational campaigns that promote healthy lifestyles should be implemented across all sociodemographic groups, particularly in educational institutions and community settings.

## Author Contributions

Conceptualisation: Killian Asampana Asosega and Eric N. Aidoo. Methodology: Killian Asampana Asosega and Eric N. Aidoo. Formal analysis: Killian Asampana Asosega and Eric N. Aidoo. Writing–original draft preparation: Killian Asampana Asosega and Eric N. Aidoo. Writing–reviewing and editing: Killian Asampana Asosega, Eric N. Aidoo, Atinuke Olusola Adebanji and Ellis Owusu‐Dabo. Supervision: Eric N. Aidoo, Atinuke Olusola Adebanji and Ellis Owusu‐Dabo.

## Funding

The authors did not receive any form of grant/funding for this study.

## Disclosure

All authors read and approved the published version of the manuscript.

## Ethics Statement

The access and use of the 2022 GDHS dataset was formally requested and approved by the DHS Program in collaboration with the ICF International Institutional Review Board (IRB) and the Ghana Health Service Ethical Committee.

## Conflicts of Interest

The authors declare no conflicts of interest.

## Data Availability

The 2022 GDHS dataset utilised in this study is publicly available in an open‐access repository of the DHS Program. The DHS datasets are generally free upon request after a formal registration process. Further details on how to access the DHS data are available at https://dhsprogram.com/data/Access-Instructions.cfm.
